# Y665F variant of mouse *Stat5b* protects against acute kidney injury through transcriptomic shifts in renal gene expression

**DOI:** 10.1038/s41598-025-15812-0

**Published:** 2025-08-21

**Authors:** Jakub Jankowski, Hye Kyung Lee, Lothar Hennighausen

**Affiliations:** Present Address: Section of Genetics and Physiology, Laboratory of Cellular and Molecular Biology, National Institute of Diabetes and Digestive and Kidney Diseases, US National Institutes of Health, Bethesda, MD 20892 USA

**Keywords:** STAT5B, SNP, Acute kidney injury, Kidney, Gene expression, Kidney

## Abstract

The impact of single nucleotide polymorphisms (SNP) on physiology is often underestimated. One amino acid change can result in a variety of phenotypes apparent only in response to disease or injury. Even known pathogenic SNPs have widespread effects that are currently unaccounted for. In this study, we investigated the impact of the known activating and pathogenic *Stat5b*^*Y665F*^ mutation in a renal injury context in mice carrying this variant. Using ischemia–reperfusion model of acute kidney injury, immunohistochemistry, RNA-seq and ChIP-seq, we establish the protective role of STAT5b activation in renal epithelium and showcase the shifts in transcriptomic landscape in a tissue not associated with the usual human phenotype of the *STAT5B*^*Y665F*^ mutation. Our data indicate new links between the JAK/STAT pathway and known kidney injury markers, contribute to the understanding of the sexual dimorphism of renal disease, and provide new potential targets for JAK inhibitor- and amino acid transport modulation-based therapies.

**Supplementary Information:**

The online version contains supplementary material available at 10.1038/s41598-025-15812-0.

## Introduction

Disease-associated single nucleotide polymorphisms (SNPs) are usually viewed through the lens of their most prominent phenotype. However, focusing an investigation on only one disease, organ, or tissue, results in overlooking their effect on the whole system and their contribution to a range of physiological responses. Further, transcriptional patterns shift significantly in any pathological setting, often putting additional strain on the altered protein’s activity. Thus, limiting inquiry to the most common symptoms is more often than not short-sighted.

Single missense mutation can significantly alter protein’s structure, and, albeit with exceptions, most proteins are not restricted to one tissue^1–3^. Transcription factors, proteins directly interacting with DNA and regulating gene expression, are usually ubiquitous. Any introduced amino acid change can result in widespread consequences, even if they are semi-tissue-restricted. For example, the same intronic SNP of the HNF1b transcription factor, mostly expressed in the kidney and pancreas, can enhance susceptibility to type-2 diabetes development, but also increase the risk of prostate cancer, and missense mutations in TBX5, usually associated with cardiomyocytes, can result in Holt-Oram syndrome and abnormal upper limb development^4–6^.

STAT transcription factors are not an exception. They perform diverse functions, both in homeostasis and disease, usually as a result of their phosphorylation due to extracellular stimuli, like cytokines. Point mutations in STATs are linked to a variety of phenotypes, spanning all the major organ systems^7–11^. Several mouse models harboring STAT mutations have been developed, establishing their role as crucial for body function. For example, STAT1 knockouts are extremely susceptible to infection and die within days after birth if not in housed in pathogen-free conditions, while STAT3 knockouts are not viable^12,13^. Multiple STAT knockout mouse strains have been investigated, but very few of them try to recapitulate gene variants observed in human disease. Among them, the *mut-Stat3* strain mimics human hyper-IgE syndrome and the STAT3 G421R strain displays T-cell dysregulation aiming to mimic primary immune deficiencies^14,15^. We have recently developed a *Stat5b* mutant mouse line, in which we introduced a SNP changing tyrosine to phenylalanine in position 665 (Y665F)^16,17^. This mutation has been detected in multiple leukemia patients and was hypothesized to be pathogenic due to STAT5B hyperactivation^18,19^. While the literature shows increased phosphorylation of STAT5B^Y665^^F^ compared to wild type *in vitro* and points to it as a driver of aberrant T-cell expansion, our previous work with this mouse strain confirms it with a demonstration of enhanced STAT5b binding to its DNA motif and higher STAT5b phosphorylation of cytokine-stimulated T-cells^16^. The mice, while displaying a number of hematopoietic abnormalities, did not directly develop malignancies. They did, however, show severe differences in the development of mammary glands, stemming from altered enhancer activity and downstream gene expression, among other physical abnormalities. There are only a few pieces of evidence for *Stat5b* being a key injury response regulator. Because of the heterogeneity of both the cellular components of the kidney and renal injury etiologies, its role is inconclusive. While impaired JAK/STAT5 signaling contributes to the development of polycystic kidney disease and chronic kidney disease, podocyte STAT5 ameliorates focal segmental glomerulosclerosis^20–22^. Only one poster publication indicates a protective role of STAT5 in an acute, cisplatin-induced injury model^23^.

In this manuscript, we follow up on our previous studies to investigate the extent of the transcriptomic shifts caused by the *Stat5b*^*Y665*^^*F*^ mutation in an acute kidney injury (AKI) setting. There is no previously published data available indicating *Stat5b’s* involvement in renal ischemic injury, providing us with an opportunity to both establish its importance in AKI, and to investigate the extent of one SNP’s effect on the transcriptional landscape of the renal epithelium. To accomplish that, we use a well-established mouse ischemic injury model, followed by immunohistochemistry and RNA-seq analysis, as well as an investigation of our previously published ChIP-seq data. Our analysis strongly indicates that STAT5b activity regulates the severity of renal injury through multiple mechanisms, such as modulating inflammation, sex-specific gene expression and amino acid transport.

## Methods

### Mice

*General care.* All animals were housed in the same environmentally controlled room (22–24 °C, with 50 ± 5% humidity and 12 h/12 h light–dark cycle) and handled according to the Guide for the Care and Use of Laboratory Animals (8th edition). All animal experiments were approved by the Animal Care and Use Committee (ACUC) of National Institute of Diabetes and Digestive and Kidney Diseases (NIDDK, MD) under the NIDDK animal protocol K089-LGP-20, and all methods were performed in accordance with the relevant guidelines and regulations as well as reported in compliance with the ARRIVE guidelines. All mice were 12–16 weeks old at the time of respective experiments. Founder mice of the *Stat5b*^*Y665*^^*F*^ strain (Y665F) were purchased from and generated at the National Heart, Lung, and Blood Institute’s Transgenic Core as described previously, by introducing the desired amino acid change via CRISPR/Cas9 and oligonucleotide template in C57BL/6N mice (Charles River Laboratories)^16^. Due to disease phenotypes requiring euthanasia at 2–3 months of age present in homozygous mice, heterozygous mutants were used. Euthanasia was performed by CO_2_ inhalation followed by cervical dislocation.

*Ischemia–reperfusion surgery.* Randomized litters of heterozygous and wildtype-bred mice were used for all the procedures. We chose approximately 2–3 months old mice to balance avoiding mortality in our severe model (3.7% or 1/27 for data presented), while aiming for more pronounced injury than in young animals. To perform warm renal ischemia–reperfusion, in random order and with the surgeon blinded to genotype, but not sex, mice were anesthetized with ketamine/xylazine mix (100 mg/kg and 10 mg/kg respectively). Hair was removed from the mouse retroperitoneal area using sterilized electrical clippers and skin was cleared and prepared using betadine and ethanol swabs. Next, the mice were placed over temperature-controlled heating pad maintained at 38 °C. Core temperature of the mice was sustained at approximately 35.5–36 °C as measured by a rectal probe. Renal Ischemia was induced by clamping the renal artery for 30 min bilaterally. Then, the clamp was removed, and skin was closed using sterile wound clips, which were removed at the time of euthanasia. Finally, the animals were injected with 1 ml saline to replenish fluids, provided analgesia (sustained release Buprenorphine, 1 mg/kg), and allowed to recover in a cage heated to approximately 37 °C until anesthesia wore off. Sham-operated animals underwent the same procedure excluding renal artery clamping.

### Renal injury assessment

Serum creatinine was measured with a colorimetric kit (Diazyme) according to manufacturer’s instructions. For histology assessment, kidneys were fixed in 10% neutral buffered formalin for 24 h, washed and stored in 70% ethanol. Preparation of paraffin slides and H&E staining was performed by Histoserv. Keyence BZ-9000 microscope was used to take serial, randomized photographs of renal cortex and outer medulla at total 400 × magnification. At least 6 photographs were taken per animal, visualizing both kidneys to ensure uniform injury. Photographs with at least 90% tissue coverage were then overlaid with a randomized point grid and percentage of injured tubules was quantified. Approximately 350 tubules per animal were assessed. Tubular dilation, loss of nuclei or membrane integrity, and protein casts were the positive injury criteria.

### Bulk RNA sequencing (total RNA-seq) and data analysis

Bulk RNA was extracted from whole frozen renal tissue from wild-type and mutant mice and purified with RNeasy Plus Mini Kit (Qiagen, 74134). Ribosomal RNA was removed from 1 μg of total RNAs, and cDNA was synthesized using SuperScript III (Invitrogen). Libraries for sequencing were prepared according to the manufacturer’s instructions with TruSeq Stranded Total RNA Library Prep Kit with Ribo-Zero Gold (Illumina, RS-122-2301), and paired-end sequencing was done with a NovaSeq 6000 instrument (Illumina). Read quality control was done using FastQC (Babraham Bioinformatics) and Trimmomatic^24^. RNA STAR was used to align the reads to mm10 genome. HTSeq and DeSeq2 were used to obtain gene counts and compare genotypes^25,26^. Genes were categorized as significantly differentially expressed with an adjusted p-value below 0.05. Differentially expressed genes were visualized with ComplexHeatmap R package, and were categorized using GSEA/Gene Ontology^27,28^.

### Western blot

Kidneys and mammary glands (positive control) were suspended in lysis buffer (50 mM Tris–Cl pH 8.0, 150 mM NaCl, 0.5% Na-DOC, 1% NP-40, 0.1% SDS, 5 mM EDTA, 1 mM PMSF, and protease inhibitor cocktail), and homogenized for 10 s using mechanical homogenizer (IKA). Solution was centrifuged at 4 °C for 15 min at 10.000 rpm and the supernatant was transferred to another tube. Protein concentration was assessed using Micro BCA Protein Assay Kit (Pierce). 20ug of protein was separated on a 4–12% Novex gradient gel (Invitrogen) for 2 h at 15 mA and transferred to a PVDF membrane (Invitrogen) in cold conditions, 1.5 h at 100 V. Membranes were blocked for 1 h with 5% nonfat dry milk in TBS-T buffer (TBS containing 0.1% Tween 20) and cut at approximately 55 kDa line as indicated by protein ladder before incubating with antibodies to avoid cross-reactivity. Membranes were incubated overnight at room temperature with the primary antibody while mixing. Following antibodies were used: STAT5B (Thermo Fisher Scientific, #13-5300), phosphor-STAT5 (Cell Signaling, #9351S) and GAPDH (Santa cruz, sc-47724). After washing, membranes were incubated for 1 h with HRP-conjugated secondary antibodies (Thermo Fisher Scientific, #31457 and #31462). Labeled protein bands were detected using SuperSignal™ West Pico PLUS Chemiluminescent Substrate (Thermo Fisher Scientific) and Amersham Imager 600 (GE healthcare). Full and unedited photographs are available as Supplementary Fig. 7 and were deposited in a public repository (see Data Availability).

### Immunohistochemistry

Kidneys were fixed in 10% neutral buffered formalin for 24 h, washed and stored in 70% ethanol. Preparation of unstained paraffin slides was performed by Histoserv. Slides were rehydrated by washing twice in xylenes, 1:1 xylenes-ethanol solution, twice in 100% ethanol, 95% ethanol, 70% ethanol, 50% ethanol, twice in water; each washing step lasting 3 min. Next, slides were boiled in Antigen Unmasking Solution (Vector Laboratories) for 10 min and incubated in fresh 3% hydrogen peroxide for 10 min. After washing with water, blocking in 2.5% goat serum (Vector Laboratories) was performed for 1 h at room temperature, after which the sections were incubated in primary antibody detecting mouse STAT5b (Invitrogen #13-5300), phospho-STAT3 (Cell Signaling #9145) diluted 1:200 in serum, or in serum alone (secondary antibody control) overnight at room temperature. Next, slides were washed twice in water and suspension of the secondary antibody was added (Goat anti-mouse IgG, Vector Laboratories, MP-7452 or goat anti-rabbit IgG, Vector Laboratories, MP-7451). After an hour of incubation at room temperature, slides were washed, and DAB substrate (Vector Laboratories) was added for 10 min. After thorough washing with water, hematoxylin solution (Sigma-Aldrich) was added for 3 min, followed by another wash. Slides were dehydrated by following the reverse order of initial washes, ending with xylenes and subsequent mounting with Permount (Fischer Chemical). Additionally, CD3 staining was performed by Histoserv Inc., using anti-mouse CD3 antibody (Cell Signaling Technology #78588). To quantify CD3-positive Keyence BZ-9000 microscope was used to take serial photographs of renal tissue at total 200 × magnification. At least 4 photographs with at least 90% tissue coverage were assessed for each animal, and number of CD3-positive cells per microscope field was averaged per animal.

### Statistics and reproducibility

GraphPad PRISM 10 was used to analyze experimental data. Normal distribution test (Shapiro–Wilk) was performed before assessing statistical significance of the findings by using appropriate measure detailed in each figure description. Tukey’s method was used for multiple comparison correction. All tests used two-tailed p-value and statistical significance was set at *p* < 0.05. Levels of statistical significance were described on graphs as follows: *p < 0.05, **p < 0.01, ***p < 0.001, ****p < 0.0001. Group sizes are reported in detail in figure descriptions. Error bars on graphs represent standard error of the mean (SEM). Any statistically analyzed data is derived from biological replicates.

## Results and discussion

### *Stat5b*^*Y665*^^*F*^ mutation protects male mice against renal injury

**Fig. 1:**
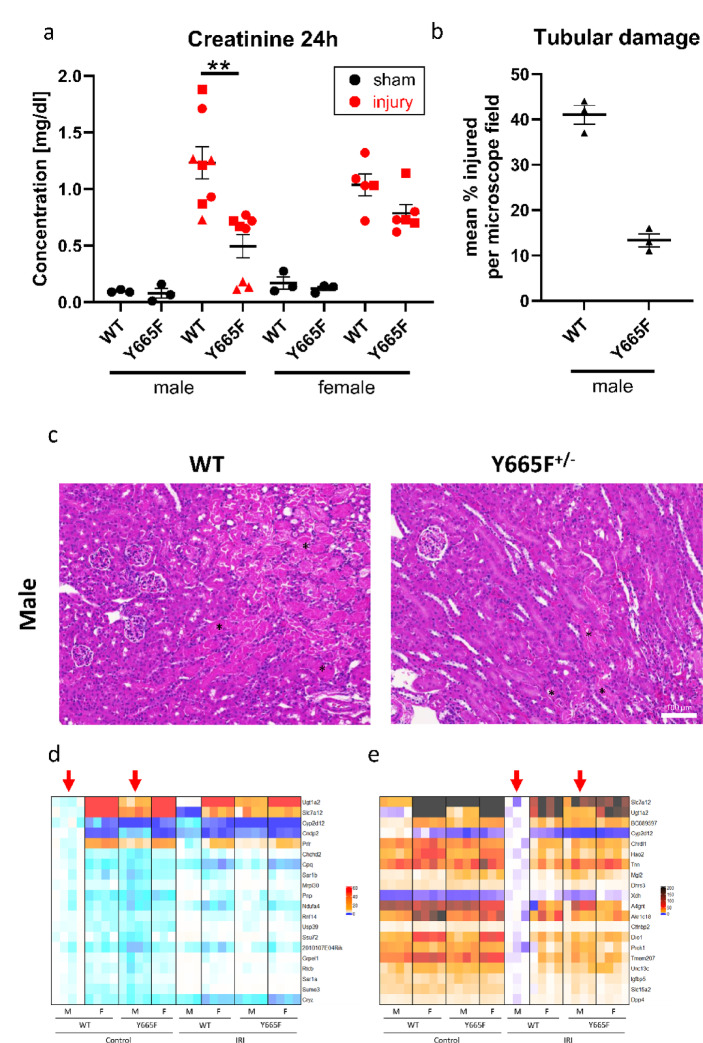
Male *Stat5b*^*Y665*^^*F*^ mutants are protected from ischemia–reperfusion injury. Serum creatinine (**a**) and tubular injury quantification (**b**) at 24 h after 30’ bilateral ischemia–reperfusion or sham surgery in male and female wild type and Y665F mice. Representative images of renal tissue from male mice 24 h after injury, with example injured tubules marked with asterisks (**c**). Heatmaps of 20 most significantly deregulated genes between male wild type and Y665F mice at the baseline (**d**) and after injury (**e**). a—n = 3 (sham groups) and n = 8 for male, n = 6 for female mice (injury groups); one WT female did not survive to the 24 h timepoint and was omitted; Two-way ANOVA with group mean comparisons between the injury groups, Bar = SEM, **p < 0.01, different symbols denote surgery cohorts; b—n = 3, average of injured tubules counted in randomized photographs; c—n = 3, bar = 100 μm, 400 × magnification; d, e—red arrows indicate columns compared for statistically significantly different gene expression.

To establish the effects of the *Stat5b*^*Y665*^^F^ mutation on renal injury, we used a 30-min bilateral ischemia–reperfusion model and euthanized mice at the 24-h timepoint. Our primary readout of the injury efficacy and response was plasma creatinine increase (Fig. 1a). We observed a smaller rise in creatinine in heterozygous mutant males compared to WT (mean 0.4948 vs. 1.231, *p* < 0.01), but not in females (mean 0.7867 vs. 1.038, *p* > 0.05). As female mice are often regarded as more protected from acute renal injury, we purposefully used a severe model to elicit a measurable response. Creatinine levels of sham-operated mice remained at the baseline and did not differ between experimental groups. Following, we investigated renal histology of male mice and confirmed the protective effect of the Y665F mutation, as fewer tubules were visibly injured in mutants than WT mice (Fig. 1b,c).

Gene expression of *Stat5b*, measured by bulk RNA-seq, did not change in the Y665F strain, not only at the baseline, which is in line with our previous observations^16^, but also in the injury setting (Supplementary Fig. 1a). We used western blot to investigate protein expression (Supplementary Fig. 1b), and detected more STAT5b protein in female mice, but without significant differences between strains and experimental conditions. Phosphorylated STAT5 signal, indicating protein activity, was uniform at the baseline and diminished after injury. We used late pregnancy mammary gland as a positive control to demonstrate significant STAT5b activation compared to the injured kidney. Additionally, immunohistochemistry revealed STAT5b-positive nuclei, but no evident differences between experimental groups (Supplementary Fig. 1c). This suggests that the germline *Stat5b*^*Y665*^^F^ mutation could be preconditioning kidneys to resist injury, rather than activating STAT5b to act acutely during ischemia and reperfusion. Expanding the investigation to the entire STAT family, we identified *Stat3* as the only other gene displaying an increase in expression after injury, smaller in mutant males compared to WT (Supplementary Fig. 2a, Supplementary Spreadsheet 1, pages 1–3). While we saw an increase in phosphorylated STAT3 after injury, once again we did not observe a clear quantitative difference between WT and mutant mice (Supplementary Fig. 2b).

Next, we investigated whether infiltrating T-cells could have a significant impact on the injury outcomes in our model. Y665F mutants, both male and female, present with splenomegaly (Supplementary Fig. 3a). CD3 antibody staining revealed significantly more T-cells present in the renal tissue before injury, but uniformly decreasing after injury (Supplementary Fig. 3b,c). This suggests that while the increased baseline T-cell presence can be to a certain degree protective in the injury setting, no abundant additional T-cell infiltration occurred at the 24 h timepoint.

In consequence, we hypothesize that epithelial STAT5b activity is the source of renal protection. We performed bulk RNA-seq to gain an overview of the renal transcriptional landscape before and after injury. The analysis revealed 187 significantly upregulated and 396 downregulated genes in between male mutant mice compared to WT males at the baseline, while 1124 were upregulated and 678 downregulated after injury. In comparison, female mice displayed no differences in gene expression between the mutants and WT mice before injury, and only six genes reached statistical significance after ischemia–reperfusion (Fig. 1d,e, Supplementary Fig. 4a, Supplementary Spreadsheet 1, pages 1–5), suggesting significant sex-dependent effect of our observations. While the following sections of the manuscript focus on the injury response, it is important to acknowledge the breadth of baseline transcriptomic changes in Y665 males (Supplementary Fig. 4). GSEA analysis strongly indicates mitochondrial involvement in this transcriptional reprogramming, and mitochondrial health has been long implicated as one of the contributors to female protection against renal injury^29^. This aligns with our observation that gene expression in male mutants is skewed towards that of females. The relative change in renal gene expression in males, compared to more stable female levels, is a likely source of the observed protective effect.

### Known injury response pathways are altered by *Stat5b*^*Y665*^^*F*^ mutation

**Fig. 2:**
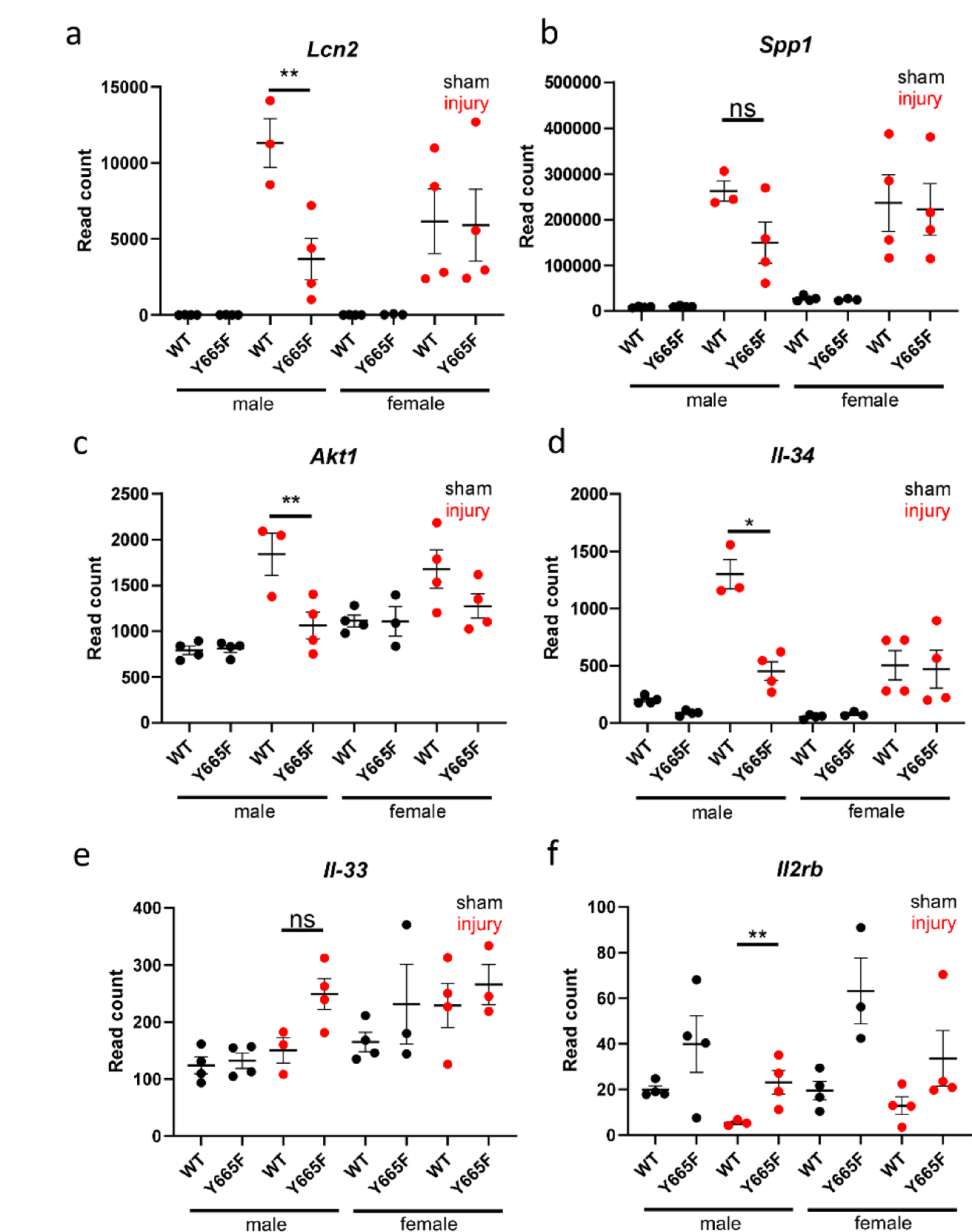
Expression of multiple injury-associated genes suggests attenuated renal injury in Y665F mutants. Normalized DeSeq2 reads of *Lcn2* (**a**), *Spp1* (**b**), *Akt1* (**c**), *Il-34* (**d**), *Il-33* (**e**) and *Il2rb* (**f**) in male and female wild type and Y665F mutants before and after injury. n = 3–4, Two-way ANOVA with group mean comparisons, Bar = SEM, *p < 0.05, **p < 0.01.

Comparing WT and mutant males after injury, there were several deregulated genes closely linked to AKI response. *Lcn2*, one of the most commonly used kidney injury markers, is significantly more upregulated in male WT than in mutant mice after ischemia–reperfusion (Fig. 2a). *Lcn2* has been reported to interact with JAK/STAT pathway through multiple mechanisms, and is usually described as anti-inflammatory in the renal injury context. It can activate NFκB/STAT3 pathway in macrophages^30^, alleviate infection through STAT1 and STAT3 downregulation^31^, but also is itself subject to upregulation through Il-1/STAT3 mechanism^32^. Its direct link to STAT5b is unknown and using previously published ChIP-seq data we were unable to find any STAT5b binding peaks lining up with GAS motifs (characteristic for STAT proteins) or H3K27ac marks designating active chromatin (Supplementary Fig. 5a).

Ischemic injury is known to activate osteopontin (*SPP1*) expression and release^33–35^. Although its expression is not statistically significantly decreased compared to WT in the injury setting (Fig. 2b), it possesses an upstream GAS motif occupied by STAT5b as evidenced by ChIP-seq analysis (Supplementary Fig. 5b). RNA-seq points to activation of downstream targets of *Spp1*, such as *Akt1* and *PIK3ca* (Fig. 2c)^36,37^. Using ChIP-seq, we observed several occupied GAS motifs in the intronic regions of *Akt1*, and smaller peaks at the promoter of the *PIK3ca*, which might be indirect binding sites, as no GAS motifs are present in the locus (Supplementary Fig. 5c, d). PI3K/AKT pathway is well-known to modulate renal apoptosis, autophagy and aging, but its link to STAT5b has not yet been established^38–40^.

JAK/STAT pathway is closely linked to cytokine stimulation and regulation of immune response. STATs are known regulators of interleukin production, while at the same time being under their transcriptomic control. For example, STAT1 regulates production of Il-1β during infection, but can also be induced by Il-2 and Il-6^41,42^, and Il-6/STAT3 axis can be targeted in multiple disease settings^43,44^. Our data indicates that STAT5b can regulate *Il-34* expression, elevated significantly higher in WT males than the mutants (Fig. 2d). Several GAS motifs and H3K27ac marks within *Il-34* locus support this conclusion (Supplementary Fig. 5e). While no strong link has been established between Il-34 and JAK/STAT signaling, there is abundant evidence suggesting that the interleukin is deleterious in ischemic setting^45–47^. Surprisingly, we did not observe the effect of the injury on the Il-33 and Il-2, known modulators of hypoxia and renal injury (Fig. 2e,f)^48–50^. While the increase in *Il2rb* in mutant males was significantly higher than that in WT males, relatively low read count, combined with lack of occupied GAS motifs observed within the ChIP-seq data, suggests that in our model this pathway does not play a significant role. However, this observation needs to take into consideration earlier reports of T-cell STAT5b activating T-cell *Il2ra* expression^51^. While more work is necessary, this could potentially reinforce the hypothesis that renal epithelium is the main source of the protective effect. Both Il-2 and Il-33 are involved in T-cell activation, and together with lack of *Stat1* gene induction, the lack of increase in expression might suggest they remain largely inert.

### Renal STAT5b drives sexually dimorphic gene expression

**Fig. 3:**
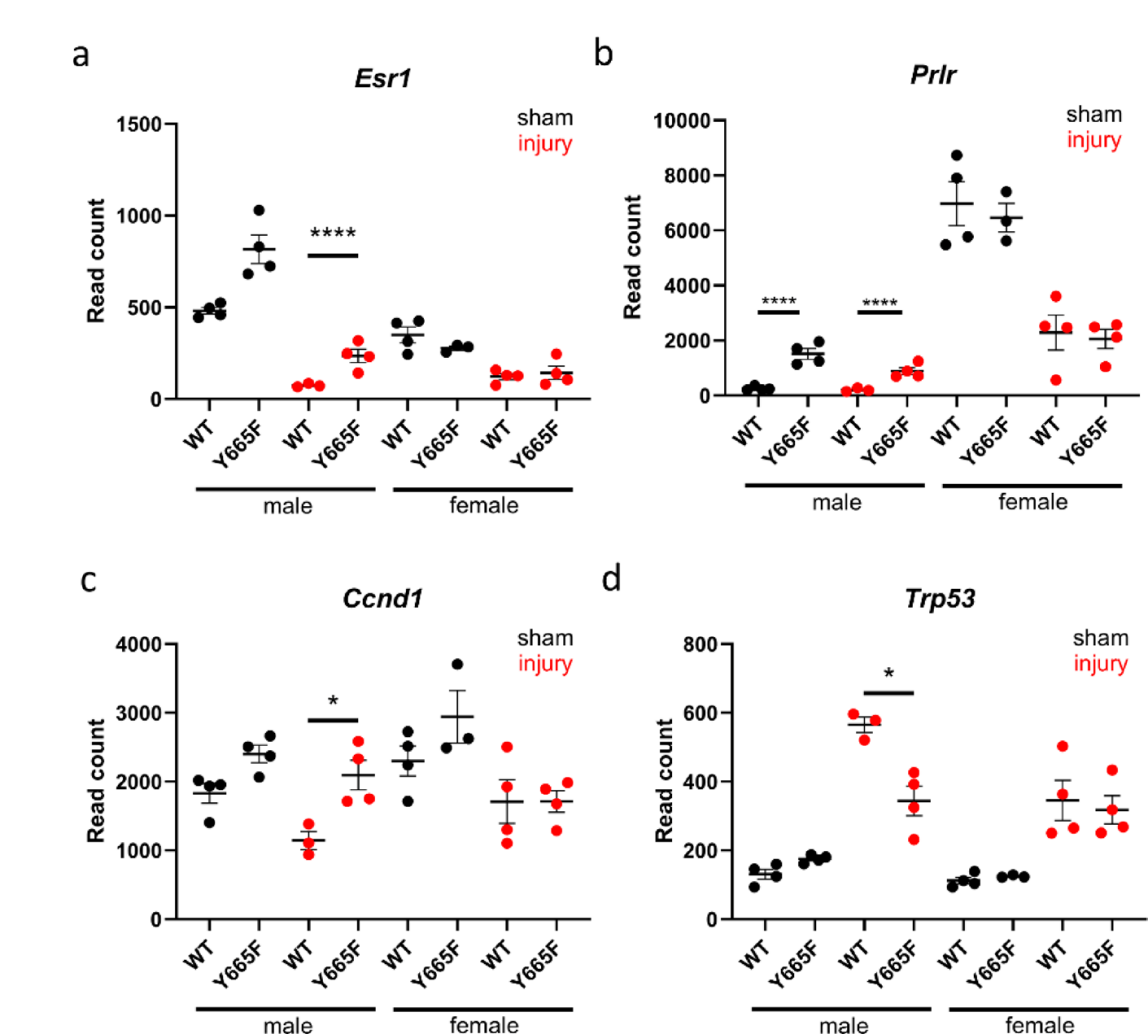
Sex hormone activity is dysregulated in Y665F males and promotes injury resistance. Normalized DeSeq2 reads of *Esr1* (**a**), *Prlr* (**b**), *CCND1* (**c**) and *Trp53* (**d**) in male and female wild type and Y665F mutants before and after injury. n = 3–4, Two-way ANOVA with group mean comparisons, Bar = SEM, *p < 0.05, ****p < 0.0001.

Response to renal disease and its progression is highly dependent on the sex of the patient^52^. It is generally recognized that men are at higher risk of AKI, while chronic kidney disease is more prevalent in women, though its progression is slower than in men^53,54^. The female protection against renal injury was proposed to diminish after menopause, strongly indicating that sex hormones play a role in the process^55^. RNA-seq results displaying the most deregulated genes between males before and after injury showcase a trend towards feminization of renal gene expression in the Y665F mutants (Fig. 1d,e). For example, *Ugt1a2* and *Slc7a12* are expressed at higher levels in male mutants, similarly to the levels found in female mice, while expression of *Cyp2d12* and *Cndp2* decreased in mutant compared to WT males. After injury this trend becomes even more obvious, as genes with elevated expression in female mice and mutant males fail to follow in male WT mice. This observation marks STAT5b activity as a driver of sexually dimorphic gene expression. A similar effect has been reported in the liver and skeletal tissue, indicating them as potential targets of follow-up studies and tissues of concern in patients harboring the *STAT5B*^*Y665*^^*F*^ mutation^56–59^. Especially the liver studies by Waxman and colleagues are a well-developed proof of the role of STAT5b in sex-specific transcriptional programing. In a recent publication, they show that adenoviral delivery of constitutively active STAT5 can regulate sexually dimorphic genes in a dose-dependent manner and feminizes the liver transcriptome, similarly to the effects observed by us in the kidneys of Y665F mice^60^. To try to discern why the sexually dimorphic effect is present, we investigated known hormone regulators present in the kidney. Estrogen receptors and sirtuins, acting in tandem, are known protective factors in renal injury and chronic disease setting^61,62^. We observed elevated *Esr1* transcript in Y665F males, but not the females, both before and after injury (Fig. 3a). Estrogen receptors can activate STAT5b through phosphorylation by JAK2^63,64^, but also a region in a small radius around *Esr1* presents several occupied GAS motifs, suggesting a feedback loop (Supplementary Fig. 6a). ESRα-STAT5b axis plays a role in mineral and water homeostasis and renal cell carcinoma development, but its role in renal injury is to be determined^65,66^. In comparison, there is little recent information about the role of prolactin in kidney disease. Prolactin receptor (*Prlr*) expression was one of the most significantly deregulated genes between male experimental groups and elevated in Y665F mutants (Fig. 3b). Interaction between prolactin and its downstream effectors like cyclin D1 and TP53 (Fig. 3c,d) is usually explored in the context of mammary tumors. Our data might indicate that in the renal injury setting, prolactin promotes cellular proliferation and regeneration^67–69^.

**Fig. 4:**
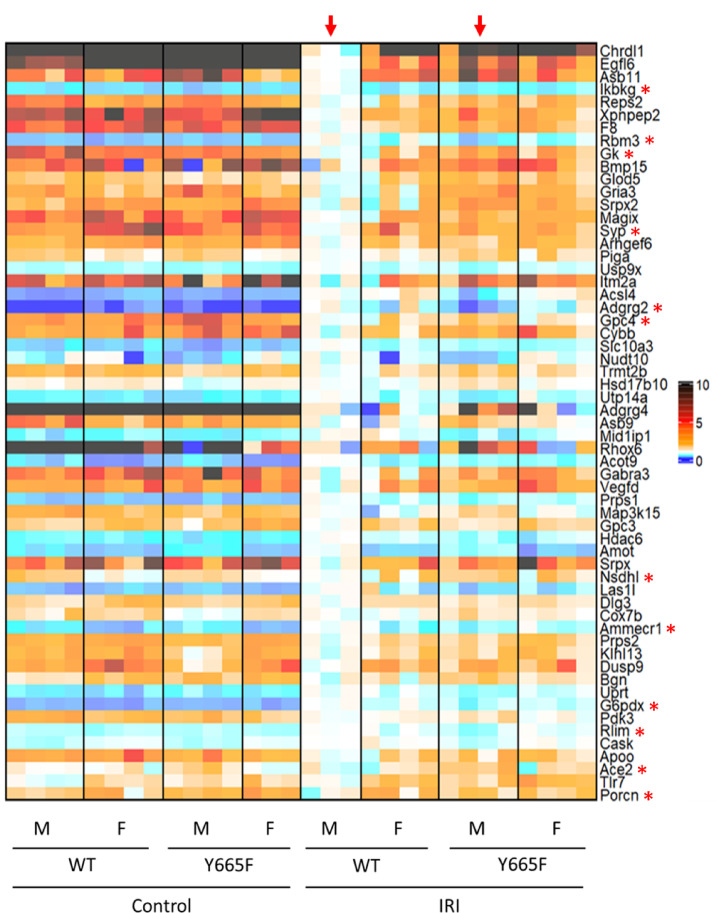
Injury causes differential expression of X-chromosome genes in wild type and mutant males. Heatmap of differentially expressed genes comparing wild type and mutant male mice after injury (arrows). Genes present on chromosome X are visualized. Asterisks indicate genes with STAT5b peaks present in ChIP-seq analysis suggesting direct modulation.

Another result of the *Stat5b*^*Y665*^^*F*^ mutation worth considering is the apparent feminization of gene expression on chromosome X (Fig. 4). There is a number of X-linked renal diseases, clinically more prevalent in men than women, and aberrant gene expression resulting from disruption of the JAK/STAT pathway is worth investigating^70^. Among 59 differentially expressed genes in male injury group, 12 possess STAT5b peaks occupying GAS motifs and thus are directly activated or inhibited by it (Supplementary Fig. 6b, c). Notably, majority of those genes does not display sexual dimorphism prior to the injury, explaining which could be the subject of a follow-up study.

### STAT5b drives renal amino acid transport

**Fig. 5:**
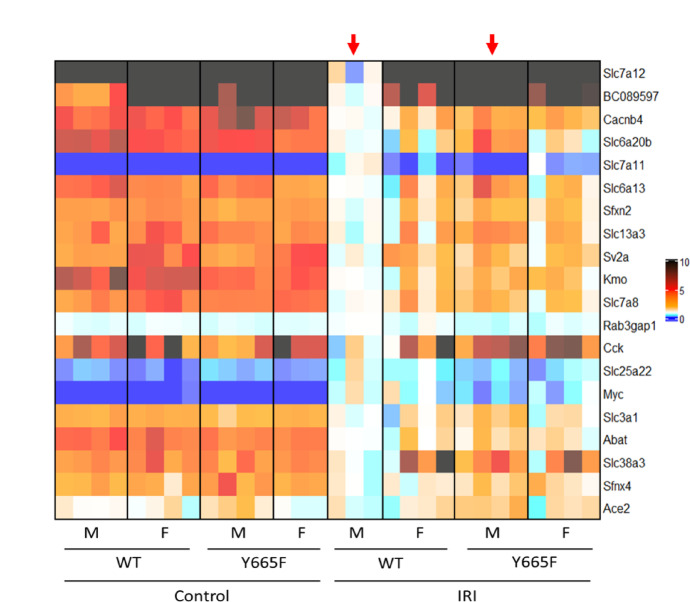
Amino acid transport in Y665F mice is altered after injury. Heatmap of differentially expressed genes comparing wild type and mutant male mice after injury. Top 20 genes categorized as taking part in amino acid transport by the GSEA Molecular Signal Database are presented.

Amino acid transport and resorption are a key function of the kidney^71^. Renal epithelium expresses a vast number of transporter proteins, most notably of the SLC family. As such, modulating and preserving these critical mechanisms has been a target of several therapeutic approaches, like inhibition of the SLC6A19 protein to attenuate renal injury^72,73^. ACE2 inhibition, often used in clinical settings, also can affect epithelial amino acid transport. Numerous SLC family members, *Ace2*, and several others were found to be significantly deregulated in male WT mice compared to mutants after injury (Fig. 5). While the JAK/STAT pathway is linked to amino acid transport, direct link to STAT5b signaling has not been established thus far^74–76^.

## Summary

In this manuscript, we have established the role of the *Stat5b*^*Y665*^^*F*^ variant as a modulator of renal injury. Attenuated elevation of plasma creatinine in mutants compared to controls, histology, and injury marker expression confirm that STAT5b activation helps protect against kidney ischemia–reperfusion model. The lack of injury-stimulated changes in *Stat5b* mRNA level and abundance of STAT5b nuclear antibody staining, as well as increased immune infiltration at the baseline, suggest prolonged presence of adaptive mechanisms allowing for the observed protection. We present several possible mechanisms explaining the findings. First, the expression levels of a number of inflammatory marker genes, including *Lcn2*, *Il-34* and *Spp1,* were lower in mutants compared to those in WT mice. Second, there is a significant sexually dimorphic component in any renal injury model, here enhanced by hyperactivated STAT5b, which skews the male renal gene expression pattern towards the female profile. Another aspect of this change is differential expression of X-chromosome genes, including ones directly under *Stat5b* control. Lastly, we show altered amino acid transporter expression, which is a potential target of already existing therapies, including those modulating ACE2 activity. Finally, our work is the first to discuss the role of STAT5b in acute ischemic injury. While JAK inhibition is used to alleviate symptoms of chronic and diabetic kidney injury, modulating STAT5B activity may be beneficial in treating the immediate injury response^77–79^.

## Supplementary Information

Below is the link to the electronic supplementary material.

Supplementary Material 1

Supplementary Material 2

## Data Availability

The datasets generated and/or analyzed during the current study are available in the NCBI GEO repository, under https://www.ncbi.nlm.nih.gov/geo/query/acc.cgi?acc=GSE289432 , https://www.ncbi.nlm.nih.gov/geo/query/acc.cgi?acc=GSE270652, and https://www.ncbi.nlm.nih.gov/geo/query/acc.cgi?acc=GSE114292, as well as in the Zenodo repository at https://zenodo.org/records/14862696. Any additional data or materials are available on request to the corresponding author (Jakub Jankowski—jakub.jankowski@nih.gov).
