# Supplementary material for: Y665F variant of mouse Stat5b protects against acute kidney injury through transcriptomic shifts in renal gene expression
Source: Sci Rep. 2025 Aug 21;15:30696. doi: 10.1038/s41598-025-15812-0 (PMC12370949; doi:10.1038/s41598-025-15812-0)
Supplement: Supplementary file 1 — Supplementary Material 1 [file 41598_2025_15812_MOESM1_ESM.pdf]

Supplementary data

Y665F variant of mouse *Stat5b* protects against acute kidney injury through transcriptomic shifts in renal gene expression

Jakub Jankowski, PhD [1,*], Hye Kyung Lee, PhD [1], Lothar Hennighausen, PhD [1]

[1] Section of Genetics and Physiology, Laboratory of Cellular and Molecular Biology, National Institute of Diabetes and Digestive and Kidney Diseases, US National Institutes of Health, Bethesda, Maryland, 20892, USA

Supplementary Figure 1

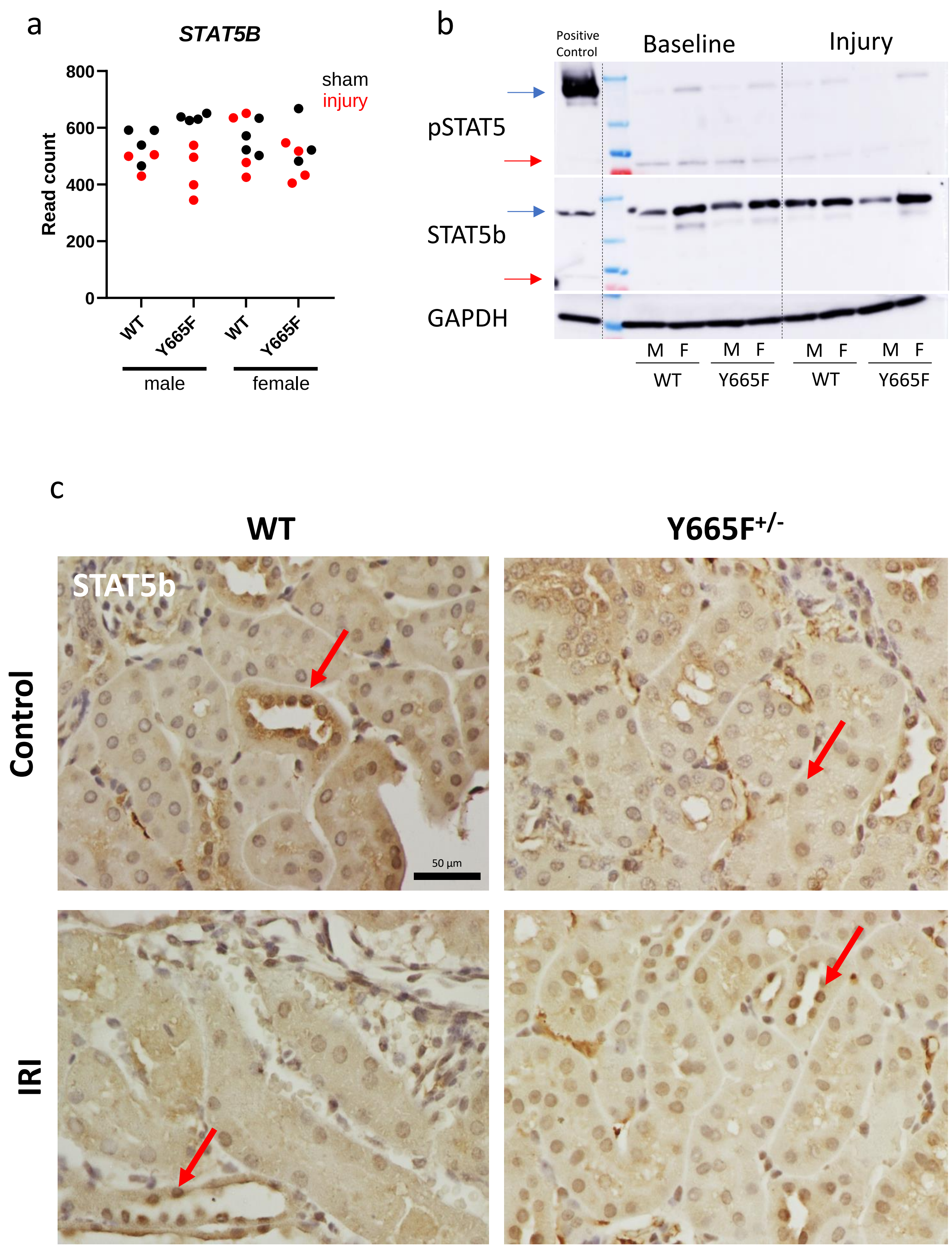

Supplementary Fig. 1 Normalized DeSeq2 reads of *Stat5b* in male and female wild type and Y665F mutants before and after injury (a), n = 3-4. Western blot of phosphorylated STAT5, STAT5b and GAPDH (36 kDa). STAT5 dimers (~180 kDa) are marked with blue, and monomers (92 kDa) with red arrow. Late pregnancy mammary gland used as positive control (b). Representative STAT5b staining images of renal tissue from male wild type and Y665F mice at the baseline and 24 hours after injury (c); bar = 50 µm, 400x magnification, arrows point to examples of positive nuclei.

Supplementary Figure 2

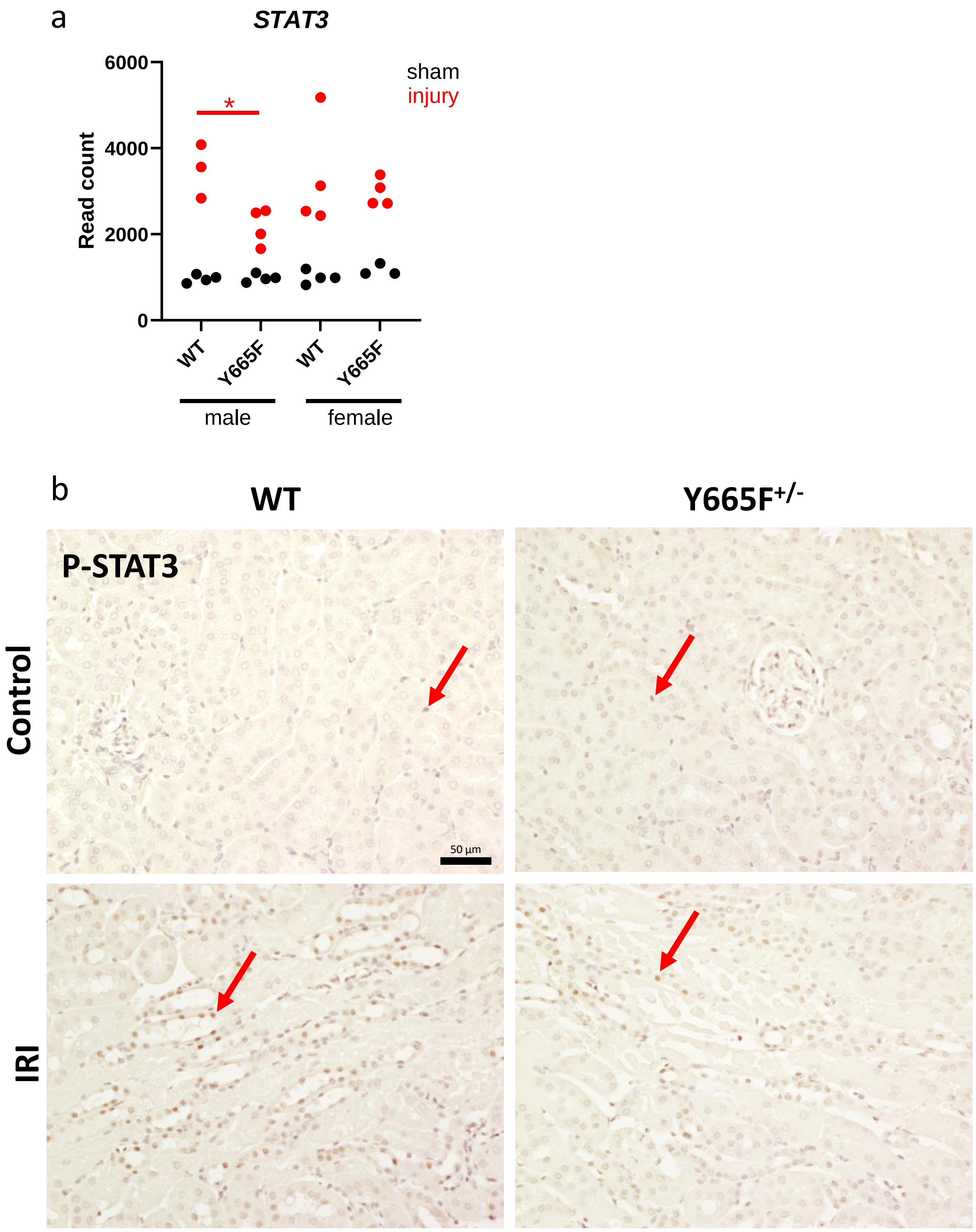

Supplementary Fig. 2 Normalized DeSeq2 reads of *Stat3* in male and female wild type and Y665F mutants before and after injury (a), n = 3-4; * $P < 0.05$. Representative P-STAT3 staining images of renal tissue from male wild type and Y665F mice at the baseline and 24 hours after injury (b); bar = 50 µm, 400x magnification, arrows point to examples of positive nuclei.

Supplementary Figure 3

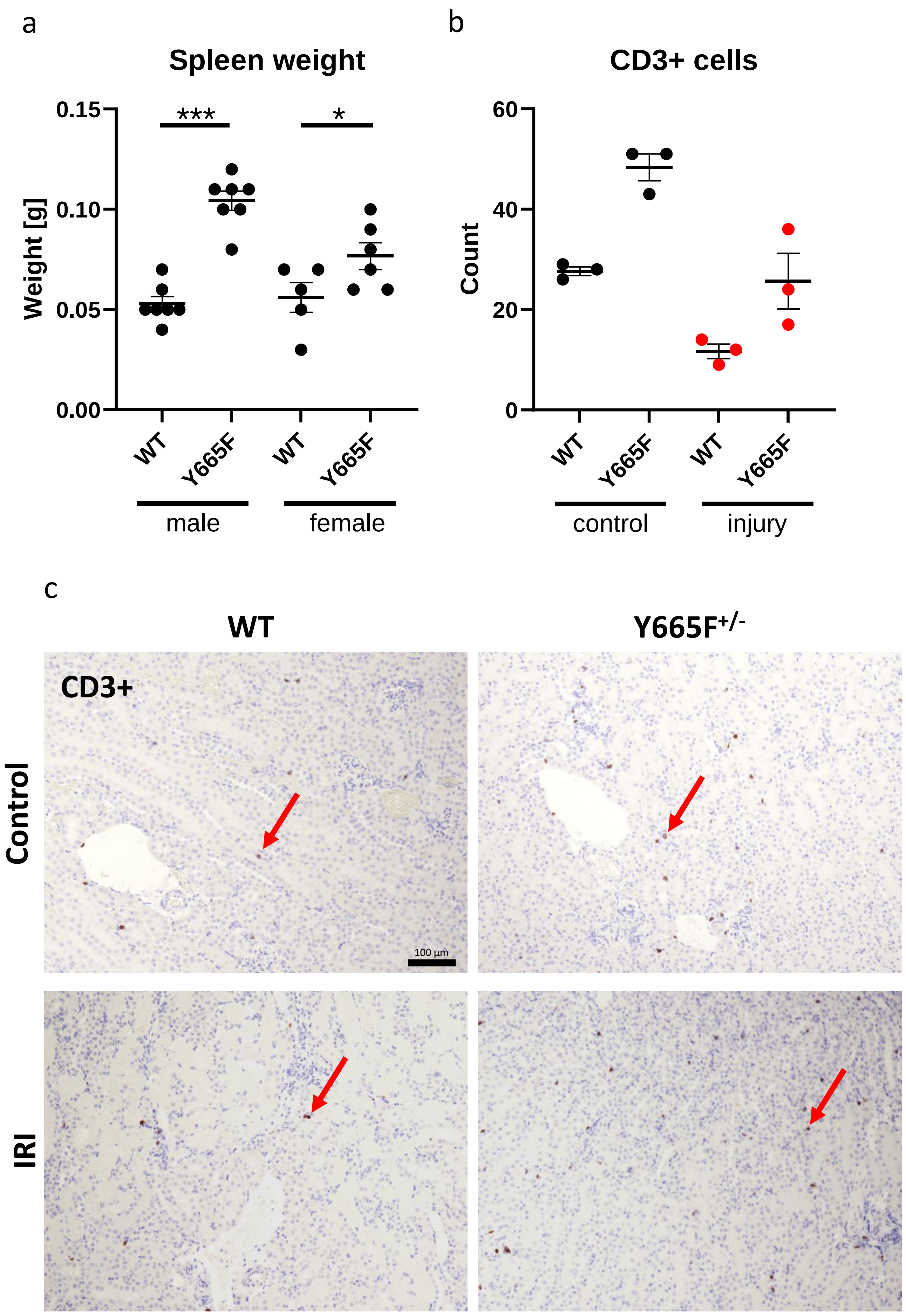

Supplementary Fig. 3 Spleen wights of male and female wild type and Y665F mice (a). n = 5-7, Bar = SEM, Two-way ANOVA with group mean comparisons, ** *P* < 0.01, *** *P* < 0.01. Number of CD3-positive cells per random microscope fields (b), n=3. Representative CD3 staining images of renal tissue from male wild type and Y665F mice at the baseline and 24 hours after injury (b); bar = 100 µm, 200x magnification, arrows point to examples of positive cells.

Supplementary Figure 4

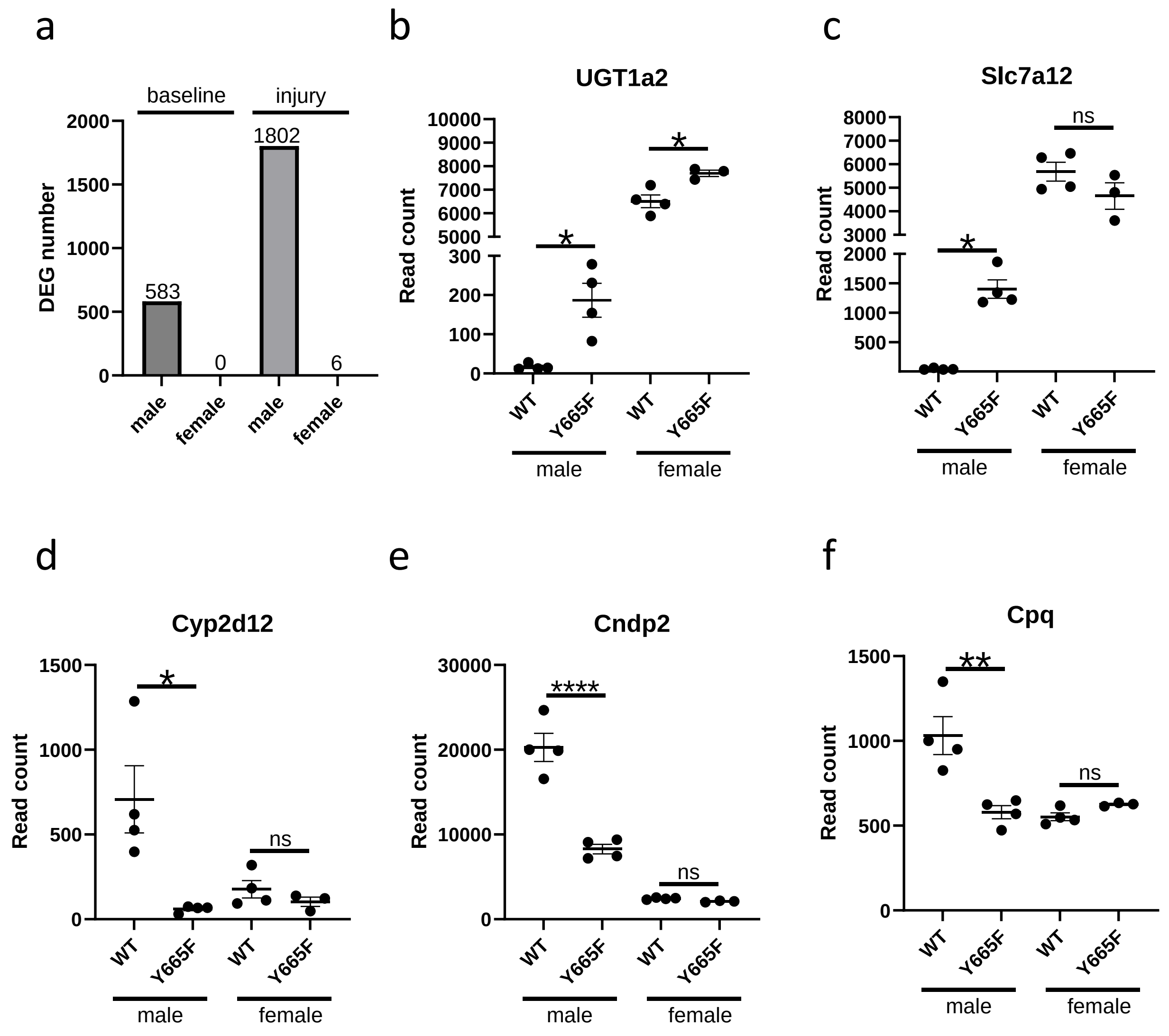

g

Gene Set Name	# Genes in Gene Set (K)	# Genes in Overlap (k)	k/K	p-value	FDR q-value
MITOCHONDRION	1912	61	0.0319	1.44E-34	1.54E-30
MITOCHONDRIAL PROTEIN CONTAINING COMPLEX	290	29	0.1	3.76E-30	2.01E-26
ORGANELLE INNER MEMBRANE	597	35	0.0586	2.99E-28	8.8E-25
MITOCHONDRIAL ENVELOPE	813	39	0.048	3.3E-28	8.8E-25
ORGANELLE ENVELOPE	1246	44	0.0353	2.21E-26	4.72E-23
SMALL MOLECULE METABOLIC PROCESS	1825	49	0.0268	3.47E-24	6.18E-21
ORGANONITROGEN COMPOUND BIOSYNTHETIC PROCESS	1690	44	0.026	3.7E-21	5.64E-18
INNER MITOCHONDRIAL MEMBRANE PROTEIN COMPLEX	158	18	0.1139	4.07E-20	5.43E-17
PURINE CONTAINING COMPOUND METABOLIC PROCESS	573	27	0.0471	1.74E-19	2.06E-16
NUCLEOBASE CONTAINING SMALL MOLECULE METABOLIC PROCESS	657	28	0.0426	4.93E-19	5.26E-16
ORGANOPHOSPHATE METABOLIC PROCESS	1012	32	0.0316	7.38E-18	7.16E-15
CATALYTIC COMPLEX	1778	39	0.0219	2.87E-16	2.55E-13
OXIDATIVE PHOSPHORYLATION	148	15	0.1014	3.46E-16	2.84E-13
RIBOSOME	268	18	0.0672	5.43E-16	4.14E-13
AEROBIC RESPIRATION	199	16	0.0804	1.42E-15	9.63E-13
GENERATION OF PRECURSOR METABOLITES AND ENERGY	493	22	0.0446	1.44E-15	9.63E-13
RIBOSE PHOSPHATE METABOLIC PROCESS	478	21	0.0439	8.93E-15	5.61E-12
ORGANOPHOSPHATE BIOSYNTHETIC PROCESS	544	22	0.0404	1.08E-14	6.4E-12
NUCLEOSIDE TRIPHOSPHATE BIOSYNTHETIC PROCESS	122	13	0.1066	1.76E-14	9.9E-12
NUCLEOSIDE TRIPHOSPHATE METABOLIC PROCESS	285	17	0.0596	2.55E-14	1.36E-11

Supplementary Fig. 4 Baseline renal gene expression changes in Y665F mice. Number of deregulated genes as indicated by RNA-seq DESeq2 analysis, each bar compares WT and Y665F mice of respective groups (a). Some of the most deregulated genes according to the RNA-seq analysis between male WT and Y665F mice at the baseline (b-f). GSEA/Gene Ontology analysis of male baseline DEGs (g). n = 3-4, Mann-Whitney or a T-test (b, c) or a Two-way ANOVA with group mean comparisons (d-f), Bar = SEM, * $P < 0.05$, ** $P < 0.01$, **** $P < 0.0001$

Supplementary Figure 5

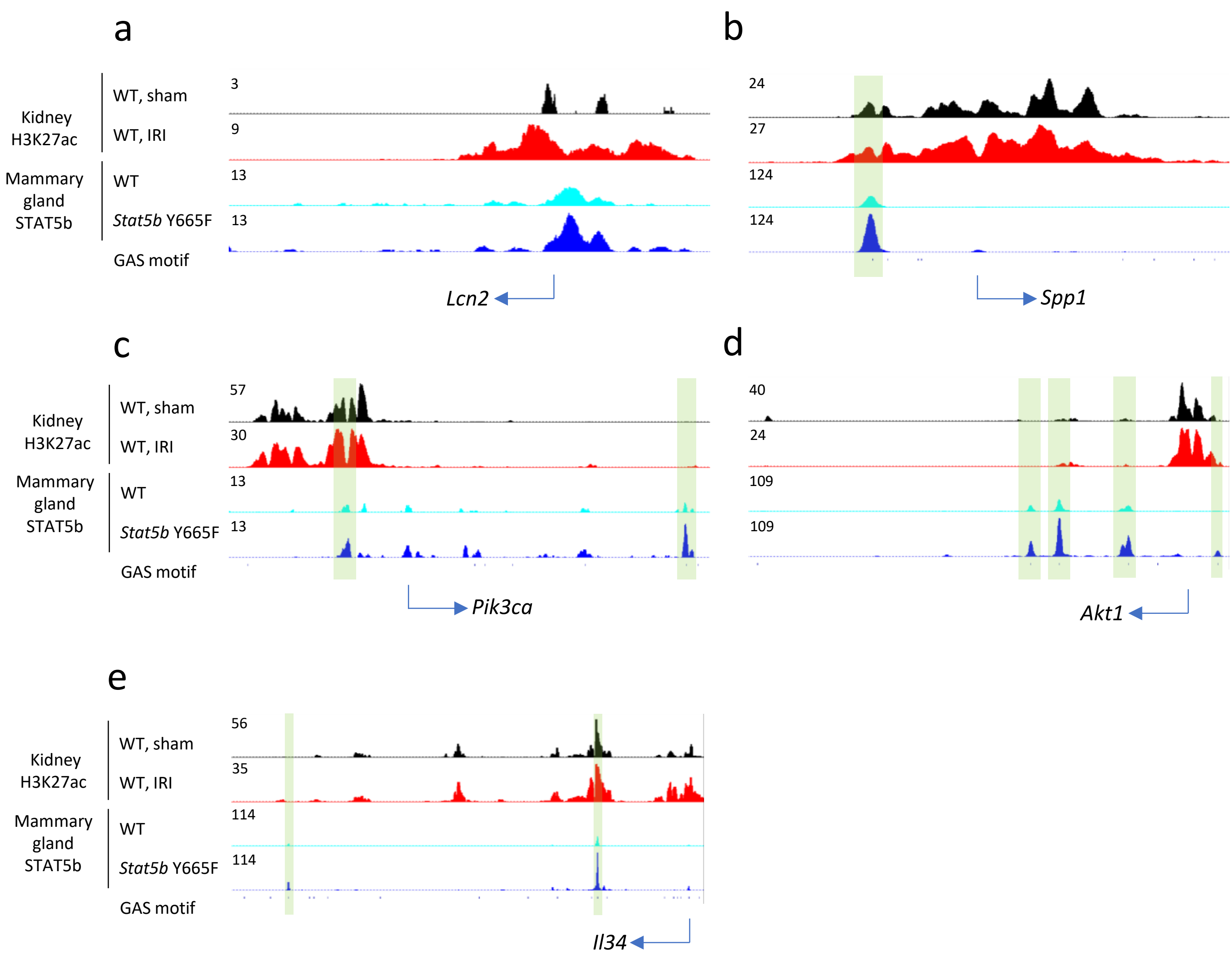

Supplementary Fig. 5 ChIP-seq tracks visualizing H3K27ac (active chromatin) in renal tissue in wild type sham and AKI mice, as well as mammary gland STAT5b binding in late pregnancy (P18) in wild type and homozygous *Stat5b*^{Y665F} mice. Relevant GAS (STAT-binding, TTCnnnGAA) motifs and potential STAT5b binding sites are marked with semi-transparent shading.

Supplementary Figure 6

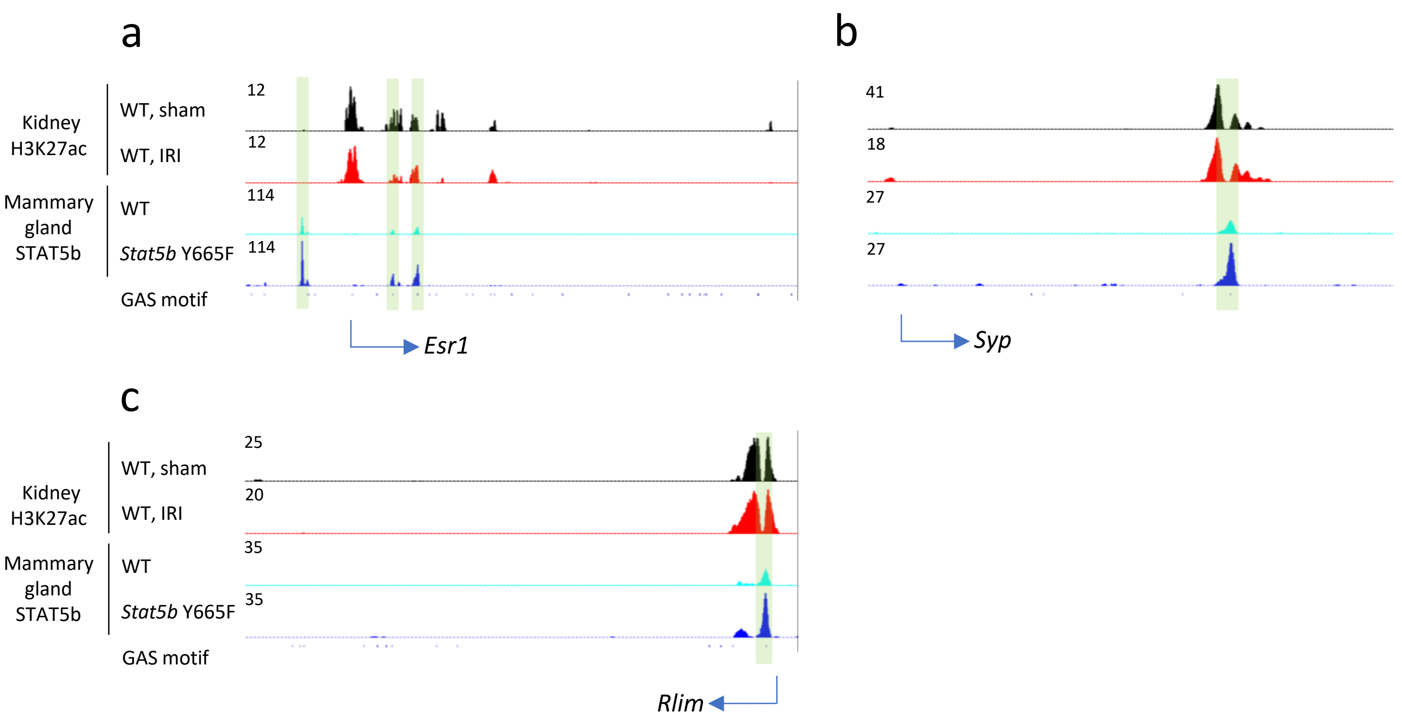

Supplementary Fig. 6 ChIP-seq tracks visualizing H3K27ac (active chromatin) in renal tissue in wild type sham and AKI mice, as well as mammary gland STAT5b binding in late pregnancy (P18) in wild type and homozygous *Stat5b*^{Y665F} mice. Relevant GAS (STAT-binding, TTCnnnGAA) motifs and potential STAT5b binding sites are marked with semi-transparent shading.

Supplementary Figure 7

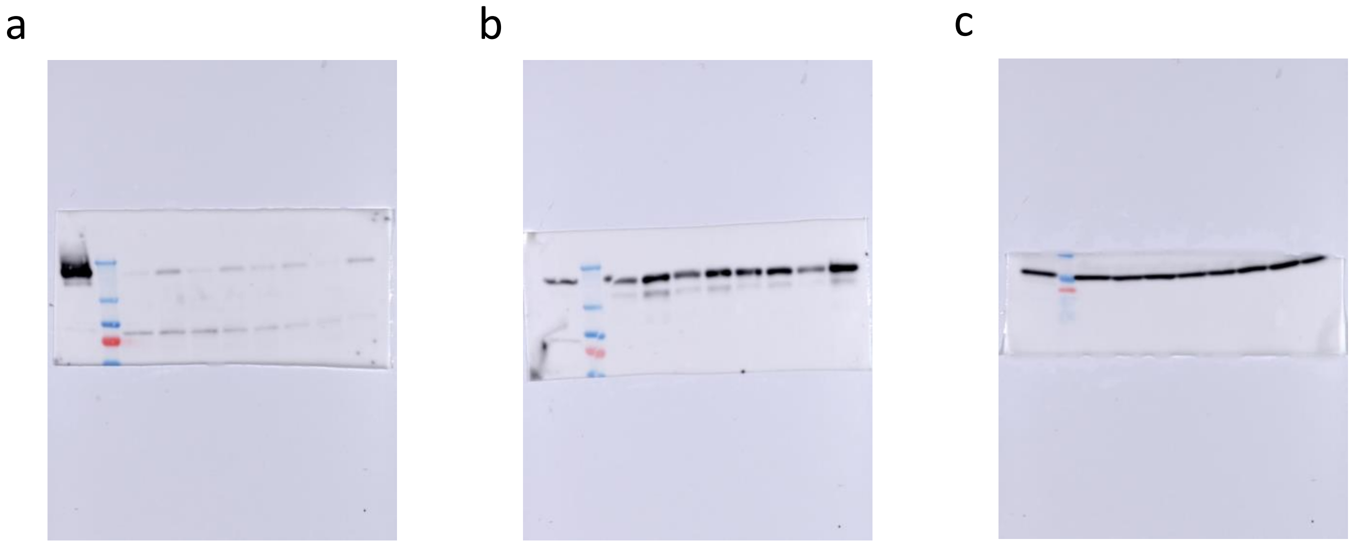

Supplementary Fig. 7 Full, unedited photographs of western blot membranes shown in Supplementary Figure 1: phosphorylated STAT5 (a), total STAT5b (b), and GAPDH (c).
